# Comparing the Effect of Dexamethasone before and after Tracheal Intubation on Sore Throat after Tympanoplasty Surgery: A Randomized Controlled trial

**Published:** 2014-04

**Authors:** Mahmoud Eidi, Seyed Javad Seyed Toutounchi, Khosro Kolahduzan, Parisa Sadeghian, Negisa Seyed Toutounchi

**Affiliations:** 1 *Department of Anesthesiology, School of Medicine, Tabriz University of Medical Sciences, Tabriz, Iran.*; 2 *Department of Otorhinolaryngology, School of Medicine, Tabriz University of Medical Sciences, Tabriz, Iran.*; 3 *Department of Anesthesiology, School of Para Medicine,TabrizUniversity of Medical Sciences, Tabriz, Iran.*; 4 *DepartmentOtorhinolaryngology School of Medicine, Tabriz University of Medical Sciences, Tabriz, Iran.*; 5 *School of Pharmacy, Tabriz University of Medical Sciences, Tabriz, Iran.*

**Keywords:** Dexamethasone, Intubation, Sore throat

## Abstract

**Introduction:**

Presence of a sore throat after surgery is a common side effect of general anesthesia with intratracheal intubation and can cause discomfort for the patient and prolong the recovery process. In this study we compared the effect of dexamethasone before and after intubation on the incidence of sore throat after tympanoplasty surgery.

**Materials and Methods:**

In a double-blind, randomized clinical trial, 70 patients aged 30–60 years with American Society of Anesthesiologists (ASA) physical status I or II who were candidates for tympanoplasty under anesthetic conditions were studied in two separate groups. The first group received intravenous (IV) dexamethasone (8 mg) 30 mins prior to intubation while the second group received the same dose of dexamethasone 30 mins after intubation. The incidence and severity of the sore throat in both groups were then evaluated.

**Results:**

There was no significant difference between two groups in intensity of sore throat (62.9% vs. 57.1%), cough (65.7% vs. 62.9%), or hoarseness (62.9% vs. 65.7%) within 24 h after surgery. Detection of blood in oral secretions or on the tracheal tube was the same in both groups (5.7%). The incidence of coughs during the extubation was 0% in first group and 11.4% in second group.

**Conclusion:**

According to the results of this research there was no significant difference in incidence and intensity of sore throat in patients receiving dexamethasone before or after intubation. Further, no significant difference in intensity of coughs or hoarseness was observed.

## Introduction

The presence of a sore throat after surgery is a very common side effect of anesthesia which can lead to discomfort for the patient after surgery and prolong recovery time (1). A sore throat following surgery can be caused by a number of factors, and the probability of each is related to the method of airway management during the operation. Thus, the prevalence of sore throat following intratracheal intubation is greatest (14.4–50%), while use of a laryngeal mask (14.4–34%) or facial mask reduces the risk to a minimum (2,3). In addition, the way in which a patient is asked has a determinative effect on the incidence of sore throat following surgery, such that after indirect questioning only two out of 129 patients complained about having sore throat while after direct questioning a total of 28 out of 113 patients made such a complaint. Such differences may be due to the fact that patients are usually more aware of symptoms that are directly related to the anesthesia and surgery rather than those not occurring immediately afterwards, such as sore throat (4). Even if the pain resulting from surgery is controlled by systematic analgesics, controlling a sore throat after surgery is difficult and therefore prevention of a sore throat is an important consideration.

It has previously been demonstrated that factors such as tube size and cuff design have a considerable effect on the probability of developing a sore throat (1). Other factors which can also cause a sore throat include pharyngolaryngeal mucosal trauma, nasogastric tube (NGT) emplacement, oral suction, the pressure affecting the tracheal capillary flow, and contact of tube and vocal cords or the internal wall of the pharynx which can cause edema and mucosal damage. An intratracheal intubation as commonly used in elective operation procedures can lead to pathologic changes, trauma, and nerve damage which can also lead to a sore throat. Thus, it seems that the pressure of the cuff is related to the nerve paralysis caused by neuropraxia. Intratracheal intubated patients report the highest rate of sore throat and other airway related symptoms (1).

Briefly, intubation causes substantial damage to the pharyngeal epithelium and trachea, even if the procedure lasts less than 1 hour. Therefore optimizing the intubation conditions and using precise techniques for decreasing the trauma is important. In most cases the pharyngeal symptoms recover spontaneously without any treatment after surgery. The most common injury is left vocal cord hematoma which has no specific cure and be meliorated spontaneously (5). In mild-to-severe sore throats and dysphagia, mouthwashes containing hydrochloride benzydamine, a non-steroidal anti-inflammatory drug with local anesthetic effect, can be useful. 

Dexamethasone is a strong corticosteroid with analgesic, anti-inflammatory, and antiemetic effects (6). It has been reported that administration of dexamethasone prior to surgery decreases the pain and edema in the oral area after surgery (7, 8). The results of a former study suggested that intravenous (IV) dexamethasone (8 mg) can be effective in reducing the incidence and severity of a sore throat after general anesthesia with orotracheal intubation (5). Although a single dose of dexamethasone is considered safe, long-term administration of corticosteroids is associated with several side effects such as glucose tolerance, increased risk of infections, delayed healing, adrenal suppression, and vascular necrosis of the bone. Dexamethasone is widely used for sore throats induced by tracheal stimulation because of its effect on edema and tissue pain (9). Furthermore, prophylactic dexamethasone is useful in decreasing airway obstruction after tracheal extubation in patients with a high risk of larynx edema (4). The painkilling mechanism may be due to anti-inflammatory effects such as inhibition of leukocyte migration, membrane stabilization, attenuation of lysosome release, and reduction of fibroblast production (4). Side effects of dexametha- sone include hyperglycemia, peptic ulcer, increased risk of infection, and electrolyte imbalance (10).

Studies reported to date principally studied the prophylactic effect of dexamethasone on sore throat after surgery compared with placebo. In this study, we compared the effect of dexamethasone, administered before and after tracheal intubation, on the incidence and severity of sore throat after surgery.

## Materials and Methods

After providing written consent, 70 adults (aged 30–60 years) with American Society of Anesthesiologists (ASA) physical status I or II who were candidates for tympanoplasty under general anesthesia in the Imam-Reza Hospital in Tabriz were selected for this double-blind, randomized clinical trial. Patients were divided randomly into two groups, each with 35 members, to receive dexamethasone before intubation (Group 1) or after intubation (Group 2). The criteria for entering the study were having ASA physical status I or II, candidate for selective tympanoplasty surgery, age 30–60 years, while exclusion criteria were recent respiratory infection or sore throat, taking steroids or painkillers before surgery, NGT, intubation time of less than 60 mins or more than 300 mins, vomiting during the study, and previous participation in an intubation trial.

Group 1 received IV dexamethasone (8 mg) 30 mins prior to intubation while Group 2 received the same dose 30 mins after intubation. All steps of the anesthesia procedure were carried out by same the anesthesiologist, like the patients, who was blinded to the randomization. The anesthetic regimen for all patients began with fentanyl (100 µg), propofol (2 mg/kg), and atracurium (0.5 mg/kg). Ventilation was applied with a 100% O2 mask before intubation. Direct laryngoscopy was applied using Mackintosh knife number 3, and tracheal intubation was applied 3 mins after injection of none-depolarized muscular relaxant (atracurium), using a low-pressure tracheal tube with an inner diameter of 7–8mm. Immediately after intubation, the tracheal tube cuff was filled under atmospheric conditions to a pressure at which no air leakage was detectable during ventilation. The cuff pressure was then set to 10–20 cm H2O manually using a barometer. The remaining anesthetic effect was attained using O2 and isoflurane (1.5%v/v) at a proportion of 1:1, while CO2 pressure at the end of the expiration was maintained at 35–40 mmHg. Following surgery, neostigmine (2.5mg) and atropine (1.25 mg) were injected in order to reverse the effect of the muscular relaxants and when inhalation and consciousness of the patient returned to normal, oral secretions was suctioned completely. After evacuation of the air from the cuff, the tracheal tube was removed and the patient received O2 through a mask prior to being transferred to the after-anesthesia care section.

Any coughs or presence of blood in oral secretions or on the tube after extubation and during the intubation was recorded. The incidence and severity of sore throat after surgery, cough, and hoarseness were evaluated by asking patients within 24 h after surgery and graded using 4-point scale chart (Table 1). The incidence and severity of sore throat after surgery were evaluated using an 11-point visual analog scale (VAS) as the primary outcome measure. In addition, the short-form McGill pain questionnaire (SFMPQ) was the main secondary outcome measure. SFMPQ (sensory) consisted of 11 components and SFMPQ (affective) consisted of four components. Patients scored each component either as none (0), mild (1), moderate (2), or severe (3) (11). Validation of the cough severity index (CSI) after surgery was evaluated using 10 questions consisting of four components 0 to 3 (12). The total dose of fentanyl or pethidine administered to induce anesthesia and during 24 h after surgery was also recorded

**Table 1:** Grading of sore throat, cough, and hoarseness severity

**Definition**	**Grade**
	Sore throat
**No sore throat** **Mild sore throat** **Moderate sore throat** **Severe sore throat**	0 1 2 3
	Cough
**No cough** **Mild cough** **Moderate cough** **Severe cough**	0 1 2 3
	Hoarseness
**No hoarseness** **No hoarseness noticed in interview but declared by patient** **Mild hoarseness** **Severe hoarseness**	0 1 2 3

In order to determine the sample size, a compare-2 means formula was used. In a former study, incidence of pain depression in a dexamethasone group was 45% compared with 10% in the placebo group (9,4). Assuming α=0.05 and 90% potency and 10% decrease in sore throat severity after surgery, the number of patients required was estimated to be 60. In order to enhance study validity, 70 patients were recruited. Random allocation software was used to group patients and the sample size was estimated using PS statistics software. 

For statistical analysis, the t-test and RAM were used to compare the age, weight, tracheal intubation duration, sore pain score, and total dose of fentanyl used during anesthesia and 24 h after surgery between the two groups. For gender and physical status (ASA) comparison, the χ²-test was used.

The difference in incidence of cough and detection of blood in oropharyngeal secretions or on the tracheal tube after extubation and the presence of sore throat after surgery, coughs and hoarseness in the 24 h after operation were analyzed using the χ²-test and Fisher exact test. These tests were also applied to the difference in severity of sore throat and coughs and hoarseness between the two groups.

The results were reported as mean ±SD, absolute number or median and interquartile range. A P-value <0.05 was considered significant. All statistical analysis was performed using SPSS 13. 

## Results

The age range of patients was 30–56 years and there was no significant difference in gender, weight, or physical status between the two groups (Table 2). Thirteen patients (37.1%) in Group 1 and 15 patients (42.8%) in Group 2 reported a sore throat 24 h after surgery (P=0.76).

**Table 2:** Demographic properties, physical status, intubation duration and cough Incidence during extubation, blood appearing in oral secretions, and fentanyl dose administered by patients in the two groups

	Group 1 (before intubation) N=35	Group 2 (after intubation) N=35	P-value
**Age**	40±7.2	40.1±8.4	0.80
**Gender (male/female)**	18/17	16/19	0.81
**Weight**	71.6±6.33	70.9±9.7	0.92
**ASA (I/II)**	34.1	26.9	0.013
**Intubation duration (min)**	107±32.5	123.2±58.1	0.22
**Cough during extubation**	(34.3)12	(37.1)13	0.36
**Blood in oral secretions**	(5.7)2	(5.7)2	
**Fentanyl dose (µg)**	20.6±92.8	26.9±80	0.06

Twelve patients in Group 1 and 13 patients in Group 2 had a cough during extubation with no significant difference between groups (P=0.36). The same result was also seen for hoarseness; 13 people in first group (37.2%) and 12 people in second group (34.3%) had hoarseness, again with no significant difference between groups (P=0.59).

No patient experienced cough during extubation in the first group while four had cough during extubation; although the difference was not statistically significant (P= 0.11) (Table 3).

**Table 3:** Incidence and severity of sore throat, cough, and hoarseness 24 h after surgery

		Group 1	Group2	P-value
	Sore throat (no.)	13 (37.1%)	15 (49.9%)	0.76
Severity	0 1 2 3	22 (62.9%) 6 (17.1%) 7 (20%) -	20 (67.1%) 7 (30%) 7 (20%) 1 (2.9%)	
	Cough (no.)	12 (34.3%)	13 (37.1%)	0.36
Severity	0 1 2 3	23 (65.7%) 11 (31.4%) 1 (2.9%) -	22 (62.9%) 9 (25.7%) 4 (11.4%) -	
	Hoarseness (no.)	13 (37.1%)	12 (34.3%)	0.59
Severity	0 1 2 3	22 (62.9%) 10 (28.6%) 3 (8.6%) -	23 (65.7%) 7 (20%) 5 (14.3%) -	

According to Table 3, there was no significant difference in severity between the two groups in patients with sore throat, cough and hoarseness.

## Discussion

Previous studies have predominantly studied the prophylactic effect of dexamethasone vs. placebo on decreasing the incidence of sore throat after surgery. In this study we compared the effect of administering dexamethasone before and after tracheal intubation on the incidence and severity of sore throat after surgery.

There are various reasons for sore throat after surgery, each of which is related to the method of airway management during the operation. According to this study, there was no significant difference between the two groups in sore throat incidence 24 h after surgery. Sore throat severity also had no significant difference between two groups.

In this study we used the same dose of dexamethasone in both groups and controlled confounding factors such as type of tracheal tube and cuff, cuff pressure, type of operation and intubation duration which could affect sore throat after surgery. Furthermore, we used same type of analgesics in both groups. Therefore the difference in sore throat severity was related only to the time of dexamethasone administration. 

Thomas et al studied on the effect of dexamethasone, administered before surgery, on sore throat and concluded that the IV dexamethasone (8 mg) reduces the incidence and severity of sore throat induced by tracheal intubation (4).

Tabari et al studied the effectiveness of betamethasone gel applied to the tracheal tube and IV dexamethasone on postoperative sore throat and showed that the incidence of sore throat was significantly lower in the betamethasone gel group compared with the IV dexamethasone and control groups at 1 h (P=0.05), 6 h(P=0.006), and 24 h(P= 0.008) postoperatively. None of the patients in the betamethasone gel group reported a sore throat 24 h postoperatively (8).

Park et al showed that prophylactic dexamethasone(0.2mg/kg)causes considerable reduction in incidence and severity of sore throat and hoarseness 1 and 24 h after tracheal tube extubation. The probable mechanism of this effect can be related to the anti-inflammatory activity of dexametha- sone which inhibits leukocyte immigration and stabilizes the cell membrane uniformity; furthermore this effect can be enhanced by administering the dexamethasone before pharyngeal trauma (10).

Wang et al studied the effect of dexamethasone on sore throat after surgery in patients undergoing thyroidectomy operation and concluded that dexamethasone decreases sore throat after surgery (13). However, unlike our study, this study did not control for confounding factors affecting sore throat after surgery such as type of tube and cuff, blood on tracheal tube after extubation, smoking history and pulmonary disease history.

In this study investigating the prophylactic effect of dexamethasone, we compared its effect when used before tracheal intubation with after intubation and concluded that there is no significant difference between two groups in the incidence and severity of sore throat. This is in contrast to the study of Park et al which showed that IV dexamethasone (10mg) before tracheal intubation is more effective in decreasing the sore throat after surgery than using it after tracheal intubation (10).

The incidence and severity of sore throat in our study was higher than expected, which may be because of the low dose of dexamethasone in comparison with other studies. Furthermore, this finding could be due to the small sample size, although statistically this sample size was sufficient. Even though former studies confirmed the prophylactic effect of dexamethasone on sore throat after surgery followed by tracheal intubation, we wanted to study on the effect of time of administration of dexamethasone. Furthermore, the effectiveness of prophylactic dexamethasone on decreasing the severity of the sore throat after surgery was verified. 

## Conclusion

There was no significant difference in reduction of severity of sore throat between groups receiving dexamethasone before and after intubation. In the group receiving dexamethasone after intubation, there was one case of severe sore throat compared with no cases in the other group. There was no statistically significant difference in incidence or severity of sore throat, coughs and hoarseness severity reduction between two groups.

## Suggestions

According to similar results in former studies with the same sample size, it is recommended that a larger sample size be used in future studies. It is also recommended that a similar study be conducted using a higher dose of dexamethasone and comparing its effect on sore throat incidence and severity. According to the results of this study, administering dexamethasone during anesthesia, without considering intubation time, is recommended.
